# The effects of symptom overreporting on PTSD treatment outcome

**DOI:** 10.1080/20008198.2020.1794729

**Published:** 2020-08-11

**Authors:** Agnes van Minnen, Birgit van Dalen, Eline M. Voorendonk, Anouk Wagenmans, Ad de Jongh

**Affiliations:** a Behavioural Science Institute (BSI), Radboud University Nijmegen, Nijmegen, The Netherlands; b Research Department, Research Department PSYTREC, Bilthoven, The Netherlands; c Academic Centre for Dentistry Amsterdam (ACTA, University of Amsterdam and VU University Amsterdam, Amsterdam, The Netherlands; d School of Health Sciences, Salford University, Manchester, UK; e Institute of Health and Society, University of Worcester, Worcester, UK; f School of Psychology, Queen’s University, Belfast, Northern Ireland

**Keywords:** malingering, treatment outcome, trauma-focused treatment, symptom overreporting, intensive treatment, simulación, resultados del tratamiento, tratamiento centrado en el trauma, sobre-informe de síntomas, tratamiento intensivo, 诈病, 治疗结果, 聚焦创伤治疗, 症状过度报告, 强化治疗, • Overreporting symptoms are often seen as an contra-indication for treatment.• PTSD-patients who overreported symptoms benefitted from trauma-focused treatment.• Overreporting symptoms may have other underlying reasons than malingering.• Overreporting patients should not be excluded from trauma-focused treatment.

## Abstract

**Background:**

It is often assumed that individuals with posttraumatic stress disorder (PTSD) who overreport their symptoms should be excluded from trauma-focused treatments.

**Objective:**

To investigate the effects of a brief, intensive trauma-focused treatment programme for individuals with PTSD who are overreporting symptoms.

**Methods:**

Individuals (*n* = 205) with PTSD participated in an intensive trauma-focused treatment programme consisting of EMDR and prolonged exposure (PE) therapy, physical activity and psycho-education. Assessments took place at pre- and post-treatment (Structured Inventory of Malingered Symptomatology; SIMS, Clinician Administered PTSD Scale for DSM-5; CAPS-5).

**Results:**

Using a high SIMS cut-off of 24 or above, 14.1% (*n* = 29) had elevated SIMS scores (i.e. ‘overreporters’). The group of overreporters showed significant decreases in PTSD-symptoms, and these treatment results did not differ significantly from other patients. Although some patients (35.5%) remained overreporters at post-treatment, SIMS scores decreased significantly during treatment.

**Conclusion:**

The results suggest that an intensive trauma-focused treatment not only is a feasible and safe treatment for PTSD in general, but also for individuals who overreport their symptoms.

## Abstract (es)

**Antecedentes**: Generalmente se asume que individuos con trastorno de Estrés Postraumático (TEPT) que sobre-informan sus síntomas debiesen ser excluidos de tratamientos centrados en el trauma.

**Objetivo**: Investigar los efectos de un programa de tratamiento centrado en el trauma breve e intensivo en individuos con TEPT que sobre-informan síntomas.

**Métodos**: Individuos (*n*=205) con TEPT participaron en un programa de tratamiento intensivo centrado en el trauma, consistente en EMDR y terapia de exposición prolongada (PE por sus siglas en inglés), actividad física y Psicoeducación. Se realizaron evaluaciones pre y post tratamiento (Inventario Estructurado de Sintomatología Simulada SIMS, Escala de TEPT para el DSM-5 administrada por el Clínico CAPS-5)

**Resultados**: Usando un corte en el inventario SIMS de 24 o mayor, 14.1% (*n*=29) tenía elevados puntajes SIMS (es decir, los ‘sobre-informadores’). El grupo de sobre-informadores mostró un descenso significativo en los síntomas de TEPT, y estos resultados de tratamiento no fueron significativamente diferentes al del resto de los pacientes. Aunque algunos pacientes (35.5%) se mantuvieron sobre-informadores posterior al tratamiento, los puntajes SIMS disminuyeron significativamente durante el tratamiento.

**Conclusión**: Los resultados sugieren que un tratamiento intensivo centrado en el trauma no es solo factible y seguro para el TEPT en general, sino que también para individuos que sobre-informan sus síntomas.

## Abstract (zh)

**背景**: 通常认为, 过度报告症状的创伤后应激障碍 (PTSD) 患者, 应被排除于聚焦创伤治疗之外。

**目的**: 探究一个简短的聚焦创伤强化治疗方案对过度报告症状的PTSD患者的效果。

**方法**: 225名PTSD患者参加了一项聚焦创伤的强化治疗方案, 包括EMDR和延长暴露 (PE) 疗法, 体育锻炼和心理教育。在治疗前, 后进行评估 (诈病症状结构量表: SIMS, 临床用DSM-5 PTSD量表: CAPS-5) 。

**结果**: 使用24或以上的高SIMS临界值, 有14.1％ (n = 29) 的SIMS得分升高 (即‘过度报告者’) 。过度报告者组表现为PTSD症状的明显减少, 并且这些治疗结果与其他患者无显著差异。虽然一些患者 (35.5％) 在治疗后仍是过度报告者, 但治疗期间SIMS得分显著下降。

**结论**: 结果表明, 聚焦创伤的强化治疗不仅是一种对于一般PTSD可行且安全的治疗方法, 对于过度报告症状的个体也是如此。

## 1. Introduction

Symptom overreporting has been associated with posttraumatic stress disorder (PTSD) in several studies, and has often been interpreted as ‘malingering’ (e.g. Hall & Hall, 2006). The prevalence of malingering among PTSD-patients (in the context of a forensic assessment) is estimated at 15% ± 15% (e.g. Young, 2015). However, some researchers have emphasized that symptom overreporting may have other underlying reasons than malingering *per se* (Merckelbach, Dandachi-FitzGerald, van Helvoort, Jelicic, & Otgaar, 2019), such as inattentive responding that may be caused by specific genuine PTSD-symptoms. For instance, difficulties with concentration, one of the key symptoms of PTSD, may lead to low attention, and thereby to inadequate answering of items on a questionnaire. Also, dissociative symptoms and alexithymia, symptoms often seen in PTSD-patients, have been found to be related with symptom overreporting (Brady, Bujarski, Feldner, & Pyne, 2017; Merckelbach, Prins, Boskovic, Niesten, & Campo, 2018). Personality traits, such as fantasy proneness have also been found to contribute to overreporting (Peace & Masliuk, 2011). The issue of the underlying cause of symptom overreporting is clinically relevant, because it may have an impact on treatment decision making. For example, if PTSD patients are caught overreporting their symptoms, this is not seldom interpreted as malingering.

Consequently, it is often recommended to exclude these patients from trauma treatment because treatment would probably be less effective and therefore not useful (e.g. Crawford et al., 2017). However, when overreporting is related to genuine PTSD-related symptoms, treatment may still be indicated and effective.

Thus far, however, little is known about the effectiveness of trauma-focused treatment regarding patients who overreport symptoms in clinical settings. In one study using veterans with PTSD (Hale, Rodriguez, Wright, Driesenga, & Spates, 2019), it was found that symptom overreporting, as measured with the Minnesota Multiphasic Personality Inventory (MMPI), was related to the severity of baseline pathology, but not to trauma-focused treatment outcome. To enhance our understanding concerning the influence of symptom overreporting on treatment outcome in clinical settings, in the present study, we assessed symptom overreporting with the commonly used Structured Inventory of Malingered Symptomatology (SIMS; Smith & Burger, 1997). In a sample of patients who underwent a brief intensive trauma-focused treatment programme for PTSD, we tested whether patients who overreported symptoms would show significantly less decrease in PTSD-symptoms associated with trauma-focused treatment compared to those who did not overreport. In addition, we explored the effects of treatment on the SIMS-scores.

## 2. Method

### 2.1. Participants

The study participants were 264 PTSD patients who were treated between August 2017 and January 2018 at the PSYTREC clinic. Inclusion criteria were: (1) being at least 18 years old, (2) having a diagnosis of PTSD established with the Clinician Administered PTSD Scale-5 (CAPS-5) for Diagnostic and Statistical Manual of Mental Disorders, 5^th^ Edition (DSM-5; APA, 2013), (3) having sufficient knowledge of the Dutch language and (4) no suicide attempts in the past three months. For 28 participants a complete baseline assessment (CAPS-5 and SIMS) was missing, for 3 patients the CAPS-5 post-treatment was missing, 2 patients dropped out prematurely, and 26 participants did not give their consent. This yielded a data sample of 205 participants, of which 73.7% was female (*n* = 151). The age range of patients at intake was 18 to 69 years, with a mean age of 39.5 years (*SD* = 12.4).

### 2.2. Procedure

The study was performed in accordance with the precepts and regulations for research as stated in the Declaration of Helsinki and the Dutch Medical Research on Humans Act (World Medical Organisation, 2001) concerning scientific research. All data were collected using the standard assessment instruments and regular monitoring outcome procedure for the PSYTREC mental health centre. In addition, the study lacked random allocation, and no additional physical infringement of the physical and/or psychological integrity of the individual was to be expected (World Medical Association, 2001).

During the two intake sessions, the inclusion criteria were checked with the CAPS-5. If the patient met the inclusion criteria, the patient was invited to sign a treatment contract and informed consent for research purposes. Between the first and second intake, patients were asked to fill out the SIMS online at home. Nine days after the last treatment day, patients returned to the treatment centre for the post-treatment assessment (CAPS-5) and were asked to fill out the SIMS online at home beforehand.

### 2.3. Treatment programme

After the intake procedure, patients started the intensive trauma-focused treatment programme for eight days. Patients received treatment for four days, after which they returned home for the weekend, and returned for another four consecutive days of treatment. During the treatment days, the patients stayed in the clinic, and each day they received individual Prolonged Exposure (PE) therapy in the morning, and individual Eye Movement and Desensitization Reprocessing (EMDR) therapy in the afternoon, both sessions lasting 90 minutes. During the rest of the day, the patients participated in physical activity and psycho-education. Therapists were trained clinical psychologists. Of note, therapists were unaware of the SIMS scores of their patients. For a more detailed description of the treatment programme, see Van Woudenberg et al. (2018).

### 2.4. Measures

#### 2.4.1. Symptom overreporting

Symptom overreporting was measured with the Dutch version of the Structured Inventory of Malingered Symptomatology (SIMS; Merckelbach, Koeyvoets, Cima, & Nijman, 2001; Widows & Smith, 2005), at pre-and post-treatment. The SIMS has 75 dichotomous items (range 0–75) that assess for exaggeration and overreporting. The SIMS has appropriate reliability and validity. Several cut-off scores have been suggested for the SIMS. To take into account that we measured symptom overreporting in a severe clinical population and symptom overreporting may be due to genuine symptoms, we used the rather high cut-off score of 24, as is recommended in previous studies (van Impelen, Merckelbach, Jelicic, & Merten, 2014; Wisdom, Callahan, & Shaw, 2010). The reliability of the SIMS in the present study was good (Cronbach’s alpha = .80 (pre-treatment) and .86 (post-treatment)).

#### 2.4.2. PTSD symptom severity

Treatment outcome was measured using the Dutch version of the Clinician-Administered PTSD Scale (CAPS-5; Boeschoten et al., 2018) a structured clinical interview with adequate psychometric properties. Interviewers were not aware of the SIMS scores of the participants.

#### 2.4.3. Comorbid psychiatric disorders

The Dutch version of the Mini-International Neuropsychiatric Interview (MINI; Overbeek, Schruers, & Griez, 1999) was used to assess comorbid psychiatric disorders and suicidal ideation at baseline.

### 2.5. Statistical analyses

IBM SPSS Statistics version 24 was used to perform the statistical analyses. An α level of .05 (two-sided) was adopted for all analyses. To test the main hypotheses, a factorial mixed ANOVA was conducted with respectively the CAPS-5 and SIMS score over time (pre- and post-treatment) as the within-subjects factor and a positive screen on the SIMS (≥ 24 vs < 24) as the between-subjects factor.

## 3. Results

### 3.1. Sample characteristics

Using a high SIMS cut-off of 24 or above, 14.1% (*n* = 29) had elevated SIMS scores, further indicated as ‘overreporters’. Patients reported multiple traumas, among those were sexual abuse (83.9%), physical abuse (82.0%), natural disasters, accidents and war (22.0%) and work-related traumas (8.8%). A high comorbidity rate was measured, as 95.1% of the sample had one or more comorbidities, and 30.2% had high suicidal risk. SIMS and CAPS-5 total scores were significantly correlated (*r*(205) = .30, *p* < .001). There were no significant differences between the two groups with regard to age, sex, trauma type, and comorbidity.

### 3.2. Effect of overreporting on treatment outcome

**Figure 1.**
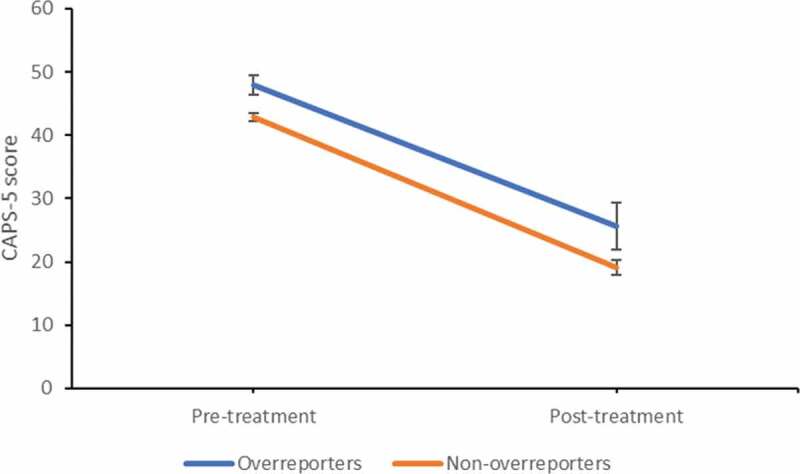
Change in mean CAPS-5 score over the course of treatment for the overreporters (*n* = 29) and non-overreporters (*n* = 176).

In Table 1 the mean CAPS-5 and SIMS total scores are depicted at pre- and post-treatment for each group. For the CAPS-5, the factorial mixed ANOVA showed a significant main effect of time, *F*(1, 203) = 233.50, *p* < .001, *η_p_^2^ *= 0.54 (large effect), and group, *F*(1, 203) = 7.36, *p* = .007, *η_p_^2^ *= 0.04 (small effect). This indicates that both groups showed large declines in PTSD symptoms over time, and that the overreporters scored significantly higher on PTSD symptoms at pre- and post-treatment than their non-malingering counterparts. No significant interaction-effect was found, *F*(1, 203) = 0.21, *p* = .65, indicating that both groups profited equally from treatment (see Figure 1).

**Table 1.** Mean (SD) scores on CAPS-5 and SIMS on pre- and post-treatment.

		Overreporters	Non-overreporters
CAPS-5	*N*	29	176
	Pre-treatment	47.90 (8.10)	42.78 (8.53)
	Post-treatment	25.62 (20.43)	19.13 (15.64)
SIMS	*N*	26	158
	Pre-treatment	30.19 (5.54), range 24–42	13.54 (4.98), range 2–23
	Post-treatment	19.73 (11.51), range 4–45	11.27 (6.77), range 1–19

SIMS = Structured Inventory of Malingered Symptomatology, CAPS-5 = Clinician-Administered PTSD scale for DSM-5.

### 3.3. Effect of treatment on SIMS scores

At post-treatment, SIMS data of 21 participants were missing, and these participants were excluded from analysis. The factorial mixed ANOVA showed a significant main effect of time, *F*(1, 182) = 86.60, *p* < .001, *η_p_^2^ *= 0.32 (large effect), and group, *F*(1, 182) = 112.10, *p* < .001, *η_p_^2^ *= 0.38 (large effect). This indicates that both groups showed large declines in SIMS symptoms over time, and that overreporters scored significantly higher on SIMS symptoms at pre- and post-treatment. A significant interaction-effect was found, *F*(1, 182) = 35.82, *p* < .001, *η_p_^2^ *= 0.16 (medium effect), indicating that the overreporters showed a stronger decrease in SIMS scores than the non-overreporters. At post-treatment, 19 patients (65.5%) did not reach the SIMS cut-off score of 24 anymore (mean 12.56, *SD* = 6.83, range 4–23), while 10 patients (35.5%) still scored above the cut-off score (mean 31.20, *SD* = 7.27, range 24–45). Although the latter group showed large effects of PTSD treatment results (CAPS Cohen’s *d* = 1.12), they showed significantly less decrease in CAPS-scores from pre- to post-treatment than the former group (*t* (27) = 2.36, *p* < .05). In the group of overreporters, the correlation between a decrease in CAPS-5 scores and SIMS scores was high (*r*(26) = .70, *p* < .001). In the total group this correlation was somewhat lower (*r*(184) = .47, *p* < .001).

## 4. Discussion

We found that in a treatment-seeking population, a part of the PTSD patients (14.1%) overreported their symptoms. Although it is often suggested that PTSD-patients who overreport should be excluded from treatment, we found that within the context of an intensive trauma-focused treatment programme, patients who overreported their symptoms showed good treatment results with large effect sizes. What is more, these treatment results did not differ significantly from other patients. Also, we found that in the group of overreporters, symptom overreporting scores declined significantly from pre- to post-treatment.

Some participants (5% of the total sample, 35% of the symptom overreporters), however, continued to overreport their symptoms at post-treatment, and these patients may have been malingerers. This percentage of malingering is in line with the literature (15% ± 15%, e.g. Young, 2015), and because we used a high SIMS cut-off score for overreporting, leaving no room for false positives (van Impelen et al., 2014), these patients are suspected of malingering. However, because we did not collect objective information about their trauma exposures and external incentives, we cannot draw this conclusion with certainty. Interestingly, although the treatment effects were lower than those of patients who did not continue to overreport their symptoms at post-treatment, these presumed malingerers also showed large treatment effects.

Patients who malinger are assumed to have external motives for pretending to have the diagnosis PTSD, and therefore it is not likely that they would respond to treatment. Several explanations might account for the present findings. As mentioned in the introduction, symptom overreporting is likely to have other underlying reasons than malingering (see for a review Merckelbach et al., 2018). Our findings showed that symptom overreporting was significantly related with PTSD-symptom severity at baseline and that the decrease of symptom overreporting was significantly and highly related to a decrease of PTSD-symptoms. Therefore, it is likely that symptom overreporting may be caused by overlapping PTSD symptoms, or to inattentive responding due to *genuine* PTSD-symptoms (or co-morbid symptoms), such as concentration problems, dissociation and depression. This is in line with general findings from different controlled PTSD-treatment outcome studies, showing that symptoms overlapping with or related to PTSD decline along with the decrease in PTSD-symptoms (see for a review van Minnen, Zoellner, Harned, & Mills, 2015).

The main limitation of our study was that it lacked a control group and randomized design. Therefore, it remains unknown to what extent the change in symptom overreporting can be attributed to our treatment. Decrease in symptom overreporting may also have been caused by regression to the mean or other confounding factors (e.g. Hawthorne effect, see McCarney et al., 2007). In addition, we used the SIMS as a standalone screening measure for overreporting, and did not include objective measures for external motives of patients. Future research should use additive performance validity tests which measure cognitive underperformance (Merkelbach et al., 2019), and gather information about patients’ external incentives. Further, our results are limited to PTSD patients in this specific clinical context, and cannot be generalized to other treatment settings, such as a forensic or military setting.

The strengths are that we included a relatively large sample size consisting of a broad range of different trauma histories. This enhances the generalization of the results as these offer a broad representation of the clinical population diagnosed with severe forms of PTSD. Another strength is that the treatment period was very brief. This makes it unlikely that, due to revictimization or other experiences during the treatment period, the underlying (external) motives for overreporting would change.

In conclusion, the present results not only suggest that trauma-focused treatment is effective for patients with severe forms of PTSD, regardless of positive scores for symptom overreporting, but also that symptom overreporting scores can decline during the course of treatment. Further, our findings show that overreporting of SIMS-symptoms alone is not per se indicative of intentional biased responding (e.g. malingering). Clearly, more research about the influence of overreporting on the treatment outcome of patients with PTSD in clinical settings is needed, including a multimodal approach that contains more valid and reliable detection measurements for assessing symptom overreporting so that likelihood of misdiagnoses can be reduced. All in all, based on the present findings excluding patients who are overreporting their symptoms from trauma-focused treatment is unjustified.
